# Food Insecurity and Health-Related Concerns Among Elementary Schoolteachers During the COVID-19 Pandemic

**DOI:** 10.5888/pcd19.210392

**Published:** 2022-05-26

**Authors:** Fangyu Li, Nivedhitha Parthasarathy, Feng Zhang, Ru-Jye Chuang, Mallika Mathur, Mike Pomeroy, Jacqueline Noyola, Christine M. Markham, Shreela V. Sharma

**Affiliations:** 1 Department of Epidemiology, Human Genetics and Environmental Sciences, School of Public Health, The University of Texas Health Science Center at Houston, Houston, Texas; 2 Department of Health Promotion and Behavioral Sciences, School of Public Health, The University of Texas Health Science Center at Houston, Houston, Texas; 3 Department of Biostatistics and Data Science, School of Public Health, The University of Texas Health Science Center at Houston, Houston, Texas; 4 Brighter Bites, Houston, Texas

## Abstract

**Introduction:**

US school systems underwent major upheaval, including closures, implementation of virtual and/or hybrid learning, and stringent infection mitigation protocols, during the initial phase of the COVID-19 pandemic. We aimed to examine the association between food insecurity and perceived health, perceived stress, and social determinants of health concerns among elementary schoolteachers serving predominantly low-income children during the COVID-19 pandemic.

**Methods:**

Brighter Bites, a nonprofit organization that weekly distributes fresh fruits and vegetables and nutrition education materials to more than 300 schools serving racial and ethnic minority populations with low income, conducts annual surveys of participating teachers to help determine subsequent efforts to support schools and families during the school year. We analyzed self-reported data collected electronically by the Brighter Bites teachers survey in 76 elementary schools during summer 2020. We used generalized linear mixed models to measure the association between food insecurity and health-related concerns.

**Results:**

Of 862 teachers who responded to the survey, 685 answered the 2 questions about food insecurity status; of these, 199 (29.1%) reported experiencing food insecurity. Food insecurity was positively associated with poor perceived general health, greater perceived stress, concerns about various social determinants of health, and changes in fruit and vegetable consumption during the COVID-19 pandemic.

**Conclusion:**

Our study demonstrated the high prevalence of food insecurity and highlights its associated factors among elementary schoolteachers during the COVID-19 pandemic. It calls attention to the high correlation of various concerns among elementary schoolteachers during the COVID-19 pandemic. Further intervention and policy efforts are needed to relieve food insecurity–related concerns and enhance well-being among teachers.

**Summary**

**What is already known about this topic?**

In the US, the prevalence of food insecurity among teachers is higher than the national average. The association between food insecurity and health-related concerns among teachers during the COVID-19 pandemic has not been investigated.

**What is added by this report?**

The prevalence of food insecurity during the COVID-19 pandemic in a survey of elementary schoolteachers was 29.1%. Food insecurity was positively associated with poor general health, greater stress, concerns about various social determinants of health, and changes in fruit and vegetable consumption during the COVID-19 pandemic.

**What are the implications for public health practice?**

A confluence of various physical, mental health, and social determinants of health concerns among elementary schoolteachers warrants attention.

## Introduction

In the US, approximately 4 million schoolteachers served approximately 56.6 million students attending public and private elementary and secondary schools during the 2019–2020 academic year (1). Compared with people in other professions, teachers earn lower wages, experience greater imbalances in effort and reward, have more occupational stress, have unhealthier eating habits, show more signs of poor physical and mental well-being, and have higher levels of food insecurity (2,3). Food insecurity is defined as the lack of nutritious foods in sufficient quantities or the disruption of eating patterns to maintain good health (4). Prior studies showed disproportionately higher rates of food insecurity among teachers than among the general US population (2,5). Additionally, studies before the pandemic showed that teachers had low levels of fruit and vegetable consumption and high intake of unhealthy foods, which puts them at increased risk for chronic conditions, such as diabetes, hypertension, and hyperlipidemia (6,7).

In 2020, the COVID-19 pandemic resulted in school closures, alterations in teaching schedules for the school year, and economic hardships (8). Teachers rapidly pivoted to virtual teaching and managing an ever-changing school environment while attending to their own health and wellness and that of their families (9). The US unemployment rate reached an all-time high of 14.8% in April 2020, putting a financial burden on nearly all people in the US (10,11). Given the national financial crisis caused by the pandemic and the higher rates of food insecurity among teachers than among the general US population before the pandemic, we sought to assess the impact of the COVID-19 pandemic on the financial stability and health of teachers. However, the literature on this topic is scarce. While previous studies demonstrated the consequences of food insecurity among preschool teachers (12,13), few assessed food-related needs among elementary schools serving racial and ethnic minority populations with low income, particularly during the COVID-19 pandemic. The purpose of our study was to describe the prevalence of food insecurity and other social determinants of health, including financial stability, employment concerns, housing, transportation, childcare, and access to health care, during the initial phase of the COVID-19 pandemic among teachers in public schools serving racial and ethnic minority families with low incomes in the US. We also explored associations between food insecurity and perceived health, perceived stress, changes in consumption of fruits and vegetables, and concerns about various social determinants of health.

## Methods

Since 2012, Brighter Bites (14), an evidence-based food co-operative program, has been implemented at more than 300 schools and summer programs in racially and ethnically diverse, low-income neighborhoods with the goal of changing behavior among children and their parents to prevent obesity and maintain long-term health. The Brighter Bites program offers weekly distributions of 50 servings of fresh, primarily donated fruits and vegetables to all parents and teachers in participating schools for 16 weeks during the school year. Schools with greater than 75% of students enrolled in the free or reduced-price lunch program are eligible for Brighter Bites. The nutrition education provided by the Brighter Bites team includes the teacher-led, evidence-based Coordinated Approach to Child Health (CATCH) program (15) in schools in Austin, Dallas, and Houston, Texas; and the District of Columbia; the Youth Understanding MyPlate (YUM) program (16) in schools in Southwest Florida; and parent education via bilingual (English and Spanish) nutrition handbooks and recipe cards. In each school year of implementation, quantitative data are collected by using a cross-sectional survey of Brighter Bites staff members, parents, and teachers. The University of Texas Health Science Center at Houston, School of Public Health, (UTHealth) is the evaluation partner for Brighter Bites and has a data-sharing agreement with Brighter Bites, a 501(c)(3) organization. The study protocol and methodology were approved by the UTHealth Committee for Protection of Human Subjects Institutional Review Board.

### Data collection

Because of pandemic-related school closures in March 2020, Brighter Bites implementation in schools came to a halt as schools turned to virtual delivery of education. Distribution of Brighter Bites food was shifted from classroom delivery to pre-prepared produce boxes for onsite pickup by families and teachers from March through August 2020. Brighter Bites operations also pivoted to support virtual implementation of the teacher-led nutrition education curriculum. UTHealth administered the Brighter Bites Teacher Survey electronically via Qualtrics (Qualtrics.XM) to all employed teachers in participating schools enrolled with Brighter Bites during the 2019–2020 school year. The purpose of the survey (37 questions) was 2-fold: first, to assess the implementation of the nutrition education activities in the schools, and second, to cross-sectionally assess the impact of the pandemic on teacher food insecurity, other social determinants of health, and perceived health and stress. This study focuses on the second aspect of the survey. The survey was sent to all 3,068 teachers across 90 Brighter Bites–participating schools starting June 15 and ending September 8, 2020, with up to 8 reminder emails per teacher for completion; 862 teachers from 76 schools responded to the survey (28.1% overall response rate). City- and region-specific response rates were 31.9% (275 of 862) in Houston, 19.1% (165 of 862) in Dallas, 27.7% (239 of 862) in Austin, 10.3% (89 of 862) in Southwest Florida, and 10.7% (92 of 862) in the District of Columbia.

**Sociodemographic characteristics.** The survey collected sociodemographic information from all survey participants, including sex, race and ethnicity, city of residence, teaching role at school, and years of experience as a teacher.

**Food insecurity.** Food insecurity was measured by using a previously validated 2-item questionnaire with a 60-day reference period (17), which included the statements “You worried whether your food would run out before you got money to buy more” and “The food you bought just didn’t last and you didn’t have money to get more.” A 3-point response scale (never true, sometimes true, and often true) was used to assess teachers’ food insecurity status. Participants who selected “sometimes true” or “often true” for either of the 2 statements were classified as food insecure, and all others were classified as food secure (17).

**Psychosocial factors and social determinants of health**. Perceived self-reported general health was assessed on a 5-point scale ranging from poor to excellent; perceived stress was measured through the question “In the last two months, due to COVID-19 and related challenges (eg, school and childcare closures, online teaching, and other personal challenges) how often have you felt nervous and ‘stressed’?”; a 5-point response scale ranged from “never” to “very often” (18). We also assessed pandemic-related social determinants of health by asking if participants had concerns about the following areas: financial stability, changes in employment status, availability or affordability of food or housing, access to transportation, childcare, or clinic/doctor. Participants reported having concerns in any area by answering yes to the yes/no question, “Due to COVID-19, are you concerned about any of the following in regard to you and your family [Choose all that apply]?” (19,20).

**Behavioral factors.** Behavioral factors, including whether teachers received Brighter Bites produce bags, were assessed by using the question “Did you receive Brighter Bites bags this year? (Weekly bags of produce).” Changes in fruit and vegetable intake were assessed both before and during COVID-19 by using 2 items: “As a result of Brighter Bites, has your intake of fruits and vegetables: increased/decreased/stayed the same as before?”, and “Due to COVID-19, has your consumption of fruits and vegetables: increased/decreased/stayed the same?”

**Participation in government assistance programs.** Teachers were asked about their participation in nutrition-related government assistance programs. The survey asked, “Does your family use the following programs [Choose all that apply]? Eg, Women, Infants, and Children (WIC), Supplemental Nutrition Assistance Program (SNAP) benefits, and free/reduced meal programs.”

### Statistical analysis

We used SAS Studio version 3.8 (SAS Institute Inc) to perform all analyses. We computed descriptive statistics, including frequencies and proportions for all sociodemographic characteristics, psychosocial factors, social determinants of health, and behavioral factors. We used χ^2^ tests to assess whether the proportions of respondents who were food insecure differed by sex, race and ethnicity, city or region, teachers’ roles at school, years working in the current position, or concerns about COVID-19–related social determinants of health and whether they received Brighter Bites produce bags, changed their consumption of fruits and vegetables as a result of COVID-19 or participation in Brighter Bites, or participated in government assistance programs. For variables that resulted in small numbers (eg, received Brighter Bites produce bags, frequency of fruit and vegetable intake before the COVID-19 pandemic, participation in nutrition-related government assistance programs), we used Fisher exact tests to determine differences by food insecurity status. Perceived general health and stress levels were ordinal variables; therefore, we used Cochran–Armitage trend exact tests to determine whether proportions were significantly different by food insecurity status. We used multilevel univariate and multivariable generalized linear mixed models (GLMMs) to determine associations between food insecurity and general health, stress level, social determinants of health concerns during COVID-19, and changes in fruit and vegetable consumption before and after adjustment for other covariates (ie, sex, race and ethnicity, and city/region). We combined the categories excellent, very good, and good into “good” and fair and poor into “not good” self-reported general health in the GLMMs. We measured food insecurity as the dependent variable in the GLMMs, whereas general health, stress level, social determinants of health concerns during COVID-19, and changes in fruit and vegetable consumption were independent variables added into the model one at a time. Because of small numbers of survey respondents who participated in government assistance programs, we did not include this variable in the GLMM. We applied repeated measures to account for school-level clustering adjusted as a random effect. Significance levels were set at *P* < .05.

## Results

Of 862 teachers who completed the survey, 685 (79.5%) responded to the 2 questions on food insecurity status and were included in analysis (Table 1). Of these, 199 (29.1%) reported experiencing food insecurity. In the overall sample, most (86.2%) teachers were female and 512 (74.8%) were from Texas. Teachers in Austin had the highest nonresponse rate (78.4%) to the food insecurity questions. Compared with other racial and ethnic groups, Hispanic teachers were the largest group (41.0%) and the largest group (51.9%) not responding to the questions on food insecurity status; Hispanic teachers also had the highest percentage (52.9%) of respondents who reported having food insecurity. We did not find other demographic differences between teachers who responded to the food insecurity questions and those who did not. Most respondents were teaching faculty (91.2%); 53.8% had fewer than 5 years of working experience in the position. Overall, 15.3% of the teachers reported being in poor or fair health. Results of the stratified analysis showed that perceived general health varied by food insecurity status; food-insecure respondents had higher rates of poor or fair health than their food secure counterparts (*P* = .03). Overall, 85.4% of teachers reported being stressed/nervous either sometimes, fairly often, or very often. In stratified analysis, the percentage of teachers who reported being stressed/nervous fairly often/very often was higher among those who were food insecure than among those who were food secure (59.3% vs 43.0%; *P* < .001).

**Table 1:** Characteristics of Teachers Participating in the Brighter Bites Teacher Survey (N = 685), by Food Insecurity Status, in 5 US Locales, 2020

Variable	No. (%)^a^			*P* value^b^
	Overall	Food secure	Food insecure	
**Food insecurity status**	685 (100.0)	486 (70.9)	199 (29.1)	—
**Sex**				
Male	93 (13.8)	58 (12.1)	35 (17.9)	.05
Female	582 (86.2)	420 (87.9)	162 (82.2)	
**Race and ethnicity**				
Black or African American	105 (16.5)	74 (16.5)	31 (16.6)	<.001
Mexican American, Latino, or Hispanic	261 (41.0)	162 (36.1)	99 (52.9)	
Other^c^	53 (8.3)	30 (6.7)	23 (12.3)	
White	217 (34.1)	183 (40.8)	34 (18.2)	
**City or region of residence**				
Austin, Texas	27 (4.0)	21 (4.3)	6 (3.0)	.64
Dallas, Texas	228 (33.3)	161 (33.2)	67 (33.7)	
Houston, Texas	257 (37.6)	175 (36.1)	82 (41.2)	
Southwest Florida^d^	82 (12.0)	62 (12.8)	20 (10.0)	
District of Columbia	90 (13.2)	66 (13.6)	24 (12.1)	
**Role at school**				
Teaching faculty	625 (91.2)	444 (91.4)	181 (91.0)	.87
Administration	60 (8.8)	42 (8.6)	18 (9.0)	
**Years working in the current position**				
<5	338 (53.8)	236 (53.6)	102 (54.3)	.89
≥5	290 (46.2)	204 (46.4)	86 (45.7)	
**Psychosocial factors**				
Self-described general health				
Poor	5 (0.7)	4 (0.8)	1 (0.5)	.03^e^
Fair	100 (14.6)	59 (12.1)	41 (20.6)	
Good	299 (43.6)	219 (45.1)	80 (40.2)	
Very good	205 (29.9)	142 (29.2)	63 (31.7)	
Excellent	76 (11.1)	62 (12.8)	14 (7.0)	
Stressed/nervous				
Never/almost never	100 (14.6)	87 (17.9)	13 (6.5)	<.001^e^
Sometimes	258 (37.7)	190 (39.1)	68 (34.2)	
Fairly often/very often	327 (47.7)	209 (43.0)	118 (59.3)	
Concerns about social determinants of health				
Financial stability	271 (39.6)	132 (27.2)	139 (69.8)	<.001
Changed employment status	163 (23.8)	89 (18.3)	74 (37.2)	<.001
Availability of food	157 (22.9)	75 (15.4)	82 (41.2)	<.001
Affordability of food	193 (28.2)	91 (18.7)	102 (51.3)	<.001
Availability/affordability of housing	87 (12.7)	38 (7.8)	49 (24.2)	<.001
Access to transportation	39 (5.7)	12 (2.5)	27 (13.6)	<.001^f^
Access to childcare	76 (11.1)	43 (8.8)	33 (16.6)	.003
Access to clinic/doctor	162 (23.6)	97 (20.0)	65 (32.7)	<.001
**Behavioral factors**				
Received Brighter Bites produce bags				
Yes	657 (95.9)	464 (95.5)	193 (97.0)	.52^f^
No	28 (4.1)	22 (4.5)	6 (3.0)	
Fruit and vegetable intake as a result of Brighter Bites				
Increased	467 (71.2)	307 (66.3)	160 (82.9)	<.001^f^
Decreased	3 (0.5)	1 (0.2)	2 (1.0)	
Stayed the same	186 (28.4)	155 (33.5)	31 (16.1)	
Fruit and vegetable consumption during COVID-19 pandemic				
Increased	221 (32.3)	144 (29.6)	77 (38.7)	<.001
Decreased	134 (19.6)	77 (15.8)	57 (28.6)	
Stayed the same	330 (48.2)	265 (54.5)	65 (32.7)	
Participation in government assistance programs				
WIC	11 (1.6)	7 (1.4)	4 (2.0)	.74^f^
SNAP benefits	8 (1.2)	2 (0.4)	6 (3.0)	.009^f^
Free or reduced-price meals at school	36 (5.3)	16 (3.3)	20 (10.0)	<.001

Abbreviations: —, does not apply, SNAP, Supplemental Nutrition Assistance Program; WIC, Special Supplemental Nutrition Program for Women, Infants, and Children.

a Not all values in categories add to value in column header because not all survey participants answered all questions. Percentages are based on number of survey participants who answered question.

b Compared food-secure participants and food-insecure participants. *P* value calculated by using χ^2^ test unless specified otherwise; significant at *P* < .05.

c Includes Asian/Pacific Islander and mixed race.

d Southwest Florida includes Immokalee.

e Cochran–Armitage trend exact test.

f Fisher exact test.

Forty percent of respondents were concerned about financial stability, and more than 20% were concerned about changed employment status, availability and affordability of food, and access to a clinic/doctor. All concerns related to social determinants of health, such as financial stability and changed employment status, significantly varied by food insecurity status. Receiving Brighter Bites produce bags was not significantly correlated with food insecurity status. Teachers with either increased or decreased fruit and vegetable intake were more food insecure than teachers whose consumption stayed the same (*P* < .001). Similarly, participation in SNAP and free or reduced-price meal programs differed significantly by food insecurity status such that a larger percentage of teachers who were food insecure (vs food secure) participated in these government assistance programs (*P* = .009 and *P* < .001, respectively).

Teachers who self-reported fair or poor general health had greater odds than teachers in good general health of having food insecurity (adjusted odds ratio [OR], 1.84; 95% CI, 1.15–2.92) (Table 2). Similarly, teachers’ stress level was also significantly and positively associated with food insecurity. Teachers who were sometimes or fairly often/very often stressed/nervous had greater odds than teachers who never/almost never felt stressed/nervous of having food insecurity (adjusted OR, 2.80; 95% CI, 1.41–5.57 for sometimes; adjusted OR, 4.74; 95% CI, 2.41–5.57 for fairly often/very often). Meanwhile, teachers with concerns about financial stability had greater odds than teachers without these concerns of experiencing food insecurity (adjusted OR, 6.29; 95% CI, 4.24–9.34). Teachers who responded yes to being concerned about changed employment status (adjusted OR, 2.46; 95% CI, 1.65–3.67), availability of food (adjusted OR, 3.54; 95% CI, 2.35–5.33), affordability of food (adjusted OR, 4.33; 95% CI, 2.91–6.44), availability and/or affordability of housing (adjusted OR, 4.11; 95% CI, 2.44–6.94), access to transportation (adjusted OR, 6.27; 95% CI, 2.92–13.47), access to childcare (adjusted OR, 2.23; 95% CI, 1.32–3.76) and access to clinic/doctor (adjusted OR, 1.93; 95% CI, 1.28–2.90) had significantly greater odds than teachers without these concerns of having food insecurity. Teachers whose fruit and vegetable consumption increased and teachers whose consumption decreased as a result of the COVID-19 pandemic had significantly greater odds than teachers whose consumption stayed the same of experiencing food insecurity (adjusted OR, 1.77; 95% CI, 1.17–2.68 and adjusted OR, 2.97; 95% CI, 1.85–4.77, respectively).

**Table 2:** Associations Between Psychosocial and Behavioral Factors and Food Insecurity Among Teachers Participating in the Brighter Bites Teacher Survey (N = 685), 5 US Locales, 2020^a^

Variables	Unadjusted GLMM model		Adjusted GLMM model b	
	OR (95% CI)	*P* value^c^	Adjusted OR (95% CI)	*P* value^c^
**Self-reported general health**				
Good (good, very good, or excellent)	1 [Reference]		1 [Reference]	
Not good (fair or poor)	1.79 (1.16–2.76)	.008	1.84 (1.15–2.92)	.01
**Stressed/nervous**				
Never/almost never	1 [Reference]		1 [Reference]	
Sometimes	2.40 (1.26–4.57)	.008	2.80 (1.41–5.57)	.003
Fairly often/very often	3.80 (2.03–7.10)	<.001	4.74 (2.41–5.57)	<.001
**Financial stability**				
No	1 [Reference]		1 [Reference]	
Yes	6.20 (4.31–8.91)	<.001	6.29 (4.24–9.34)	<.001
**Changed employment status**				
No	1 [Reference]		1 [Reference]	
Yes	2.63 (1.82–3.81)	<.001	2.46 (1.65–3.67)	<.001
**Availability of food**				
No	1 [Reference]		1 [Reference]	
Yes	3.83 (2.63–5.58)	<.001	3.54 (2.35–5.33)	<.001
**Affordability of food**				
No	1 [Reference]		1 [Reference]	
Yes	4.55 (3.18–6.53)	<.001	4.33 (2.91–6.44)	<.001
**Availability and/or affordability of housing**				
No	1 [Reference]		1 [Reference]	
Yes	3.84 (2.42–6.11)	<.001	4.11 (2.44–6.94)	<.001
**Access to transportation**				
No	1 [Reference]		1 [Reference]	
Yes	6.19 (3.06–12.50)	<.001	6.27 (2.92–13.47)	<.001
**Access to childcare**				
No	1 [Reference]		1 [Reference]	
Yes	2.04 (1.25–3.33)	.004	2.23 (1.32–3.76)	.003
**Access to clinic/doctor**				
No	1 [Reference]		1 [Reference]	
Yes	1.94 (1.34–2.81)	<.001	1.93 (1.28–2.90)	.002
**Fruit and vegetables consumption as a result of the COVID-19 pandemic**				
Stayed the same	1 [Reference]		1 [Reference]	
Increased	2.17 (1.47–3.20)	<.001	1.77 (1.17–2.68)	.007
Decreased	3.01 (1.94–4.66)	<.001	2.97 (1.85–4.77)	<.001

Abbreviations: GLMM, generalized linear mixed models; OR, odds ratio.

a Austin, Texas; Dallas, Texas; Houston, Texas; Southwest Florida (Immokalee); District of Columbia.

b Adjusted for sex, race and ethnicity, and city or region.

c *P* value calculated by using GLMM. Significant at *P* < .05.

## Discussion

Our findings showed a high prevalence (29.1%) of household food insecurity during the COVID-19 pandemic among elementary schoolteachers employed at schools participating in the Brighter Bites program. Moreover, a significant proportion of our teachers reported being stressed, having poor health, and having concerns about social determinants of health needs during this time. COVID-19 has resulted in many hardships, particularly for low-income and racial and ethnic minority groups in the US, who are historically at higher risk for food insecurity and disproportionately higher risk for negative health and economic outcomes (21). In March and April 2020, national estimates of food insecurity reached 38%, which tripled the average prevalence of food insecurity in the previous 5 years (22). During the pandemic-induced lockdown in 2020, schools in many countries closed for extended periods, and 468,800 employees in US public schools lost their jobs in April 2020 alone (23). Moreover, teachers earn 20% less than workers with similar education and experience, and up to one-quarter of teachers leave the profession every year (24). The COVID-19 pandemic could have further exacerbated financial challenges, potentially resulting in the food-related concerns we observed. Additionally, the high prevalence of food insecurity found in our study was likely further influenced by seasonality because households with children no longer had access to school meals during the summer months (25).

Our study found that food insecurity was associated with poor perceived health, greater mental stress, and concerns about social determinants of health; these findings concur with findings of previous studies (19,26). Seligman and Schillinger (27) presented a conceptual framework for how the combination of stress and food insecurity could affect health: families who are on a limited budget do not have the time and money needed for essential nutrition and medical care, which compounds the effect on health and health outcomes. Furthermore, the time and money needed to respond to health conditions further strains the household budget, causing the cycle to continue (27). Prior research demonstrated an association between the burden of chronic diseases and food insecurity (28). Numerous studies also reported that food insecurity was associated with an increased risk of adverse mental health outcomes, including generalized anxiety, stress, and posttraumatic stress disorder, both before and during COVID-19 (29,30). Food insecurity is also positively correlated with other social determinants of health, such as financial instability and changed employment status (31). Various studies, even pre-pandemic studies, demonstrated high levels of stress and burnout among US elementary schoolteachers (32). Furthermore, in the workplace, the pandemic has created stressful working conditions for teachers (33). Those results, along with the results of our study, highlight that psychosocial stress could lead to chronic absenteeism among teachers and affect the quality of education delivered at schools; the research also underscores an immediate need to implement evidence-based wellness strategies at the workplace and outside the workplace to address these concerns (33,34). Additionally, policy approaches are needed to address financial stress, such as increasing teacher salaries, establishing workplace policies to provide teachers with help in preventing or reducing stress and burnout, and providing strong professional development opportunities for all teachers across rich and poor school districts (35).

In our study, food-insecure teachers were more likely than food-secure teachers to change fruit and vegetable consumption in either a positive or negative direction. These results concur with those of recent studies that reported food-insecure respondents consumed fewer fruits and vegetables than food-secure respondents and were more likely to report decreasing their consumption after the pandemic began (36,37). Several factors may explain why fresh fruit and vegetable consumption decreased in the beginning of the pandemic, including low availability and poor quality, high price, reduced grocery shopping, and concerns about food contamination (38,39). Whether this behavioral change remained warrants investigation.

Finally, our results suggest the need for interventions to maintain teachers’ financial stability and improve their access to food and health care. Despite the strong evidence showing that participating in nutrition assistance programs improves food security as well as current and long-term health outcomes (40) and despite a high prevalence of food insecurity (29.1%) in our study population, we found that less than 10% of the teachers in our study were enrolled in a government assistance program (eg, WIC, SNAP benefits). Participation in government assistance programs such as SNAP could provide the critical access to food that teachers may need at this time. However, it is unclear whether the low participation rates reflect the lack of eligibility among our teacher population or whether other factors influence these participation rates. Results of our study underscore the need to explore these factors and improve access to these services in our teacher population. Although federally funded efforts (eg, Pandemic EBT [Electronic Benefits Transfer], summer food service programs) were deployed to assist children in continued access to affordable food, to our knowledge, no such efforts were available for schoolteachers, and as such they were not eligible to participate (41,42). Future policies should also include teachers in government assistance programs during times of crisis.

This study had several limitations. First, the Brighter Bites Teacher Survey was limited in the number of sociodemographic factors collected because of the main purpose of the survey, an interest in preventing a burden on respondents, and the nature of sensitive information. We did not collect data on several factors, such as household income, that would further our understanding of food insecurity status (43). Second, in this cross-sectional study, we collected responses from the survey at one point in time, which precludes causal interpretations. Moreover, the snapshot does not represent the long-term behavior of teachers; we were unable to assess any longitudinal behavior changes. To address this limitation to a certain extent, we included questions on “prior to school closure due to COVID-19” or “due to COVID-19” in half of the questions about fruit and vegetable consumption. Third, only a subgroup of teachers employed at schools implementing Brighter Bites completed the survey; therefore, findings from the convenience sample of teachers in our study may not be generalizable to the Brighter Bites teacher population or all elementary schoolteachers in the US. However, we had a 100% response rate at the school level.

Our study described the high prevalence of food insecurity and its associated factors, including poor general health, psychosocial stress, social determinants of health, and health behaviors among elementary schoolteachers during the COVID-19 pandemic. Future studies could further explore teachers’ experiences during the pandemic longitudinally and the need to provide institutional support to mitigate food insecurity and enable well-being among teachers.
